# Improvement of a fermentation process for the production of two *Pf*AMA1-DiCo-based malaria vaccine candidates in *Pichia pastoris*

**DOI:** 10.1038/s41598-017-11819-4

**Published:** 2017-09-20

**Authors:** Robin Kastilan, Alexander Boes, Holger Spiegel, Nadja Voepel, Ivana Chudobová, Stephan Hellwig, Johannes Felix Buyel, Andreas Reimann, Rainer Fischer

**Affiliations:** 1 Fraunhofer Institute for Molecular Biology and Applied Ecology IME, Forckenbeckstraße 6, 52074 Aachen, Germany; 2 Institute for Molecular Biotechnology, Worringerweg 1, RWTH Aachen University, 52074 Aachen, Germany; 3 Indiana Biosciences Research Institute, 1345W. 16th St. Suite 300, Indianapolis, IN 46202 USA

## Abstract

*Pichia pastoris* is a simple and powerful expression platform that has the ability to produce a wide variety of recombinant proteins, ranging from simple peptides to complex membrane proteins. A well-established fermentation strategy is available comprising three main phases: a batch phase, followed by a glycerol fed-batch phase that increases cell density, and finally an induction phase for product expression using methanol as the inducer. We previously used this three-phase strategy at the 15-L scale to express three different AMA1-DiCo-based malaria vaccine candidates to develop a vaccine cocktail. For two candidates, we switched to a two-phase strategy lacking the intermediate glycerol fed-batch phase. The new strategy not only provided a more convenient process flow but also achieved 1.5-fold and 2.5-fold higher space-time yields for the two candidates, respectively, and simultaneously reduced the final cell mass by a factor of 1.3, thus simplifying solid–liquid separation. This strategy also reduced the quantity of host cell proteins that remained to be separated from the two vaccine candidates (by 34% and 13%, respectively), thus reducing the effort required in the subsequent purification steps. Taken together, our new fermentation strategy increased the overall fermentation performance for the production of two different AMA1-DiCo-based vaccine candidates.

## Introduction

The yeast *Pichia pastoris* was initially used for the production of single-cell protein, with the C1 compound methanol as a carbon source^1^, but is now widely exploited as an expression host for a broad range of recombinant proteins^2,3^. The success of *P*. *pastoris* reflects its ability to combine the advantages of two other important expression systems: the bacterium *Escherichia coli*, and mammalian cell lines such as Chinese hamster ovary (CHO) or human embryonic kidney (HEK) cells^4^. Like *E*. *coli*, *P*. *pastoris* is capable of rapid growth (doubling times of 1–3 h)^5^ and achieves high cell densities (>130 g L^−1^ dry cell weight) in inexpensive defined media^1^ while producing recombinant proteins at milligram-to-gram levels^3,5,6^. However, unlike *E*. *coli*, it also provides the eukaryotic machinery for posttranslational modification, and therefore allows the efficient secretion of correctly folded proteins^5^. This is a key advantage of highly purified proteins because *P*. *pastoris* secretes only a few host cell proteins into the fermentation broth.

Although sophisticated *P*. *pastoris* fermentation strategies have been developed to maximize product yields, including mixed carbon source feeds^7,8^ and growth-controlled feed profiles during the induction phase^9,10^, process development has been simplified by the *Pichia* Protocols^11,12^ and the comprehensive *Pichia* handbooks available from Thermo Fisher Scientific (formerly under the Life Technologies brand Invitrogen), including the well-known “*Pichia* fermentation process guidelines”^13^. The recommended fermentation process uses the strong inducible *AOX1* promoter to control recombinant protein expression^14,15^. The process consists of three phases (hereafter, the three-phase process) starting with a batch phase, during which cells are grown in a defined basal salt medium with glycerol as the sole carbon source. The excess glycerol represses the expression of genes driven by the *AOX1* promoter^16^. Derepression is achieved during the second phase when glycerol is fed in a limiting manner to increase the cell density^17^. Finally, recombinant protein expression is induced by the growth-limiting addition of methanol.

Although widely used, one major drawback of this strategy is its aim to generate high cell densities (up to 220 g wet cell weight [WCW] per liter) before the induction phase is initiated, while avoiding oxygen limitation during the entire process. If the dissolved oxygen tension (DOT) cannot be maintained above 20%, the guidelines recommend the addition of pure oxygen to the gas line or, if possible, the application of gauge pressure to the reactor.

In small-scale fermentation runs, almost all of the suggested ways to manipulate the DOT are feasible, including very high agitation rates to increase the oxygen transfer rate (OTR). But at least two of the aforementioned strategies for increasing the DOT/OTR are either impractical or too expensive at the production scale. High agitation rates can be applied in laboratory bioreactors but not in production-scale reactors because the power consumption increases by a power of five with scale at a constant energy dissipation rate^18,19^ and is normally limited to 5 kW m^−3^ ^18^. Furthermore, the process-scale addition of pure oxygen is expensive and special precautions are required because the use of compressed oxygen is accompanied by safety issues.

Nevertheless, we previously used the three-phase strategy to express another malaria vaccine candidate (PIMP) and its variants^20^. However, we have continuously optimized the fermentation process for new vaccine candidates^21^. Optimization resulted in a fermentation strategy that was still based on the “*Pichia* fermentation process guidelines”^13^ but the following modifications were introduced to avoid the aforementioned limitation in OTR: (i) a reduced glycerol concentration in the basal salt medium (20 g L^−1^); (ii) a prolonged glycerol fed-batch phase, increasing the recommended 4 h^13^ to a maximum of 20.5 h in order to compensate for the reduced glycerol in the basal salt medium and to avoid any oxygen limitation during this stage; and (iii) a methanol-limited induction phase. With these modifications and a headspace gauge pressure of 1 bar, we were able to achieve high cell densities while maintaining the DOT above 30% throughout the process without the addition of pure oxygen. These measures resulted in the successful expression of three malaria vaccine candidates (VAMAX1, VAMAX2 and VAMAX4)^21^ and a straightforward scalable process without the need for special precautions that are normally required when working with pure oxygen.

Despite the improvements, the modified process still had one major drawback that emerged during the prolonged glycerol fed-batch phase of 18.5–20.5 h, which was necessary to reach the recommended cell density of 180–220 g L^−1^ ^13^. The *AOX1* promoter is tightly repressed in the presence of excess glycerol^20–22^ and no target protein is produced during this phase, so the modification resulted in an unfavorable space-time yield.

We therefore optimized the fermentation process further and ultimately arrived at a strategy that comprises only two phases, a batch phase and an induction phase. Although the induction phase is initiated at a much lower (~25%) cell density than the equivalent stage in the three-phase strategy, the process is not only more convenient for the operator and easier to scale up, but it also generates higher product yields with a simultaneously lower level of process-related impurities.

## Results and Discussion

Our previously reported expression of the three malaria vaccine candidates VAMAX1, VAMAX2 and VAMAX4^20^ was based on the fermentation strategy in the “*Pichia* fermentation process guidelines”^13^ modified by us as described above. The modifications reduced the high oxygen demand that typically accompanied the standard three-phase fermentation strategy, but at the expense of a reduced space-time yield. We therefore decided to omit the glycerol fed-batch phase in favor of a two-phase fermentation strategy comprising a batch phase directly followed by an induction phase. Such a simplified strategy had already been used successfully for the intracellular production of the Hepatitis V virus surface antigen with *P*. *pastoris* ^22^.

To ensure the comparability of the two different strategies, we extended the induction phase of the two-phase process to compensate for the duration of the omitted glycerol fed-batch phase. Thus, the total process time was adjusted to that of the three-phase process (71 ± 2 h). An oxygen-limited induction mode with a constant methanol concentration of 2.5 mL L^−1^ was chosen for both strategies.

We compared the former three-phase fermentation strategy with the new two-phase process for two of the vaccine candidates: VAMAX1 and VAMAX2 (Supplementary Fig. S1). A fusion protein similar to VAMAX2 but lacking the *Pf*s25 fusion antigen has already been expressed in *P*. *pastoris* by Faber *et al*.^23^. The fermentation process was optimized further^24^ using a highly automated production plant integrating a design-of-experiments concept^25,26^

For all cultivations we used a modified basal salts medium (mBSM) and a modified *Pichia* trace metals (mPTM) solution^20^. The concentrations of all salt components in the mBSM was 80% lower than the standard fermentation medium^13^ and the mPTM solution contained less copper and cobalt. The mBSM/mPTM combination had no impact on the cell growth or the final cell density^27^ but improved the yield of another malaria vaccine candidate produced at our institute (data not shown).

### Cell growth and process reproducibility

**Figure 1:**
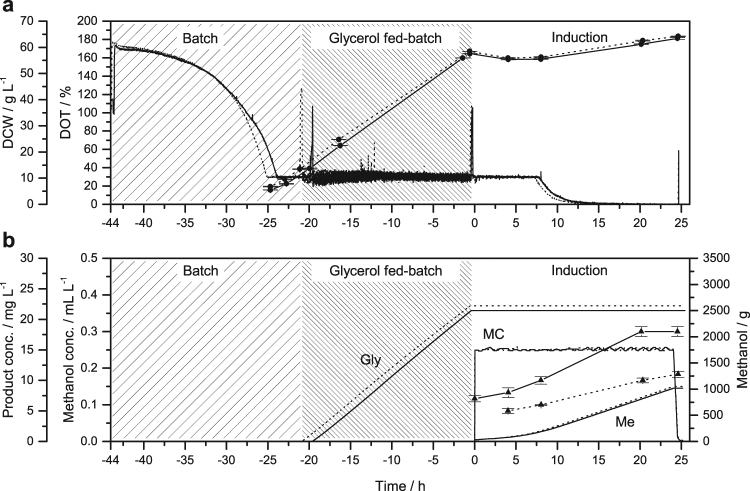
Time course of the three-phase fermentation strategy for the production of VAMAX1 (straight lines) and VAMAX2 (dotted lines) at the 15-L scale. The strategy included a typical glycerol fed-batch phase to increase the cell density before induction. An oxygen-limited induction mode with a constant methanol concentration of 2.5 mL L^−1^ was used for product expression. (**a**) Course of dry cell weight (DCW, •) and dissolved oxygen tension (DOT). (**b**) Course of added glycerol (Gly) and methanol (Me), methanol concentration (MC), and product concentration (▴). The x-axis is normalized to the time point of induction. Error bars indicate the standard deviation of technical triplicates.

In the three-phase process, the batch phase was followed by a glycerol fed-batch phase during which the glycerol solution was supplied at a constant rate. This achieved similar cell densities for both candidates, as determined by measuring the dry cell weight (DCW): 57.6 ± 0.1 g L^−1^ (n = 3) for VAMAX1 and up to 58.5 ± 0.1 g L^−1^ (n = 3) for VAMAX2 (Fig. 1a). These values corresponded to WCWs of 190 ± 2 g L^−1^ (n = 3) and 200 ± 1 g L^−1^ (n = 3), respectively, and were thus in agreement with the recommended range of cell concentrations when entering the induction phase of the three-stage process^13^. We chose a feed rate that guaranteed a minimum DOT of 30% while the bioreactor was operating almost at its maximum OTR, resulting in a very low growth rate of 0.069 h^−1^.

The glycerol fed-batch phase was followed by the induction phase, during which the concentration of methanol was kept constant at 2.5 mL L^−1^ (Fig. 1b). Although neither the methanol nor the oxygen was limited, a typical drop in cell density was observed 9 h post-induction, probably reflecting starvation-dependent cell shrinkage as observed for *Saccharomyces cerevisiae* ^28^. Given the low biomass yield of cells fed on methanol and the corresponding low growth rates (Table 1), the final cell density for both candidates increased by only ~1.1-fold 24 h post-induction.

**Table 1:** Comparative fermentation data of the three-phase and two-phase fermentation strategies.

Clone/candidate	VAMAX1		VAMAX2	
Fermentation strategy	3-phase	2-phase	3-phase	2-phase
Biomass yield on methanol [g g^−1^]	0.18	0.28	0.19	0.27
Growth rate before induction [1 h^−1^]	0.069	0.179	0.069	0.193
Growth rate during induction phase [1 h^−1^]	0.007	0.030	0.007	0.031
Specific productivity [mg g^−1^ h^−1^]	0.011	0.019	0.007	0.019
Final product concentration [mg L^−1^]	18 ± 1	28 ± 1	11 ± 1	27 ± 2
Total product yield (15-L scale) [mg]	297 ± 14	474 ± 17	186 ± 8	455 ± 28
Space-time yield (induction) [mg L^−1^ h^−1^]	0.623	0.519	0.381	0.524
Space-time yield (total process) [mg L^−1^ h^−1^]	0.224	0.343	0.136	0.343

Technical standard deviations (n = 3) are shown for the final product concentrations and total product yields.

**Figure 2:**
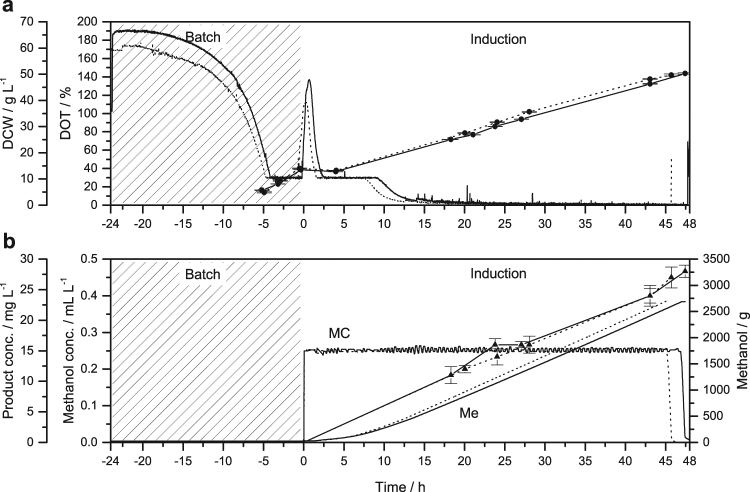
Time course of the two-phase fermentation strategy for the production of VAMAX1 (straight lines) and VAMAX2 (dotted lines) at the 15-L scale. The typical glycerol fed-batch phase was omitted in this strategy, allowing the direct transition to an induction phase after the depletion of the batch glycerol. The induction time was expanded to incorporate the time usually allocated for the glycerol fed-batch phase, resulting in almost the same total process duration as the three-phase strategy. An oxygen-limited induction mode with a constant methanol concentration of 2.5 mL L^−1^ was used for product expression. (**a**) Course of dry cell weight (DCW, •) and dissolved oxygen tension (DOT). (**b**) Course of added methanol (Me), methanol concentration (MC), and product concentration (▴). The x-axis is normalized to the time point of induction. Error bars indicate the standard deviation of technical triplicates.

In the two-phase strategy, the cell density at the time of induction reached only 13.5 g L^−1^ for VAMAX1 and 14.0 g L^−1^ for VAMAX2 (Fig. 2a), corresponding to ~24% of the recommended cell density before induction. After the typical drop in DCW, the cells reached final densities of 50.0–50.6 g L^−1^ (VAMAX1) and 49.3–50.1 g L^−1^ (VAMAX2) during the induction phase with methanol kept at a constant 2.5 mL L^−1^ (Fig. 2b). Although the conditions during the induction phase of both strategies were similar, the increase in DCW was considerably higher in the two-phase strategy compared to the three-stage strategy, i.e. ~3.6-fold for VAMAX1 and ~3.7-fold for VAMAX2. This was clearly the result of a ~4-fold higher growth rate during the production phase of the two-phase process (Table 1). We can only speculate as to the cause of this variation in growth rate, but phase preceding induction must have an impact and the higher growth rate during this phase may be important: ~2.6-fold for VAMAX1 and ~2.4-fold for VAMAX2 (Table 1). *P*. *pastoris* upregulates gene expression and translation in response to higher growth rates^29,30^. The upregulated state may in turn facilitate faster adaption to methanol in the two-phase process compared to the three-phase process, where the cells must accommodate a considerably reduced growth rate during the extended glycerol fed-batch phase (~20 h).

In order to evaluate the reproducibility of each strategy, we compared the DOT and cell growth (Figs 1a and 2a). With the exception of a negligible time offset (0.5–1.0 h) both curves followed a similar course indicating that both fermentations were reproducible regardless of the product. The biomass yield on glycerol for the batch phase was 0.71–0.74 g g^−1^ with a coefficient of variation of 1.5% (n = 4). The mean value of 0.72 g g^−1^ is similar to values reported by other groups^31,32^.

Both strategies described herein achieved much lower cell densities than those typically reported for high-cell-density fermentations in *P*. *pastoris* ^33–36^. For the two-phase strategy, this mostly reflected the low cell density at the beginning of the induction phase: 13.5 g L^−1^ for VAMAX1 and 14.0 g L^−1^ for VAMAX 2. Although the ability of *P*. *pastoris* to grow to high densities is often presented as an advantage, it becomes a drawback when large-scale bioreactors rapidly reach their OTR capacity due to the large number of cells. At the laboratory scale, high agitation rates of more than 2500 rpm can be applied to achieve high OTRs of up to 0.5 mol L^−1^ h^−1^ ^37^. However, an OTR of ~0.1 mol L^−1^ h^−1^ is already regarded as high in large-scale processes, mainly due to power restrictions that typically limit the energy dissipation rate to 5 W kg^−1^ ^18^. In the two-phase process with low cell densities, such considerations became obsolete during the growth phase, thus facilitating process scale-up. In addition to the benefit of a reduced OTR during the growth phase, lower cell densities are favorable because they facilitate solid–liquid separation (the removal of cells and cell debris by centrifugation and/or filtration) thus reducing the overall process costs.

**Figure 3:**
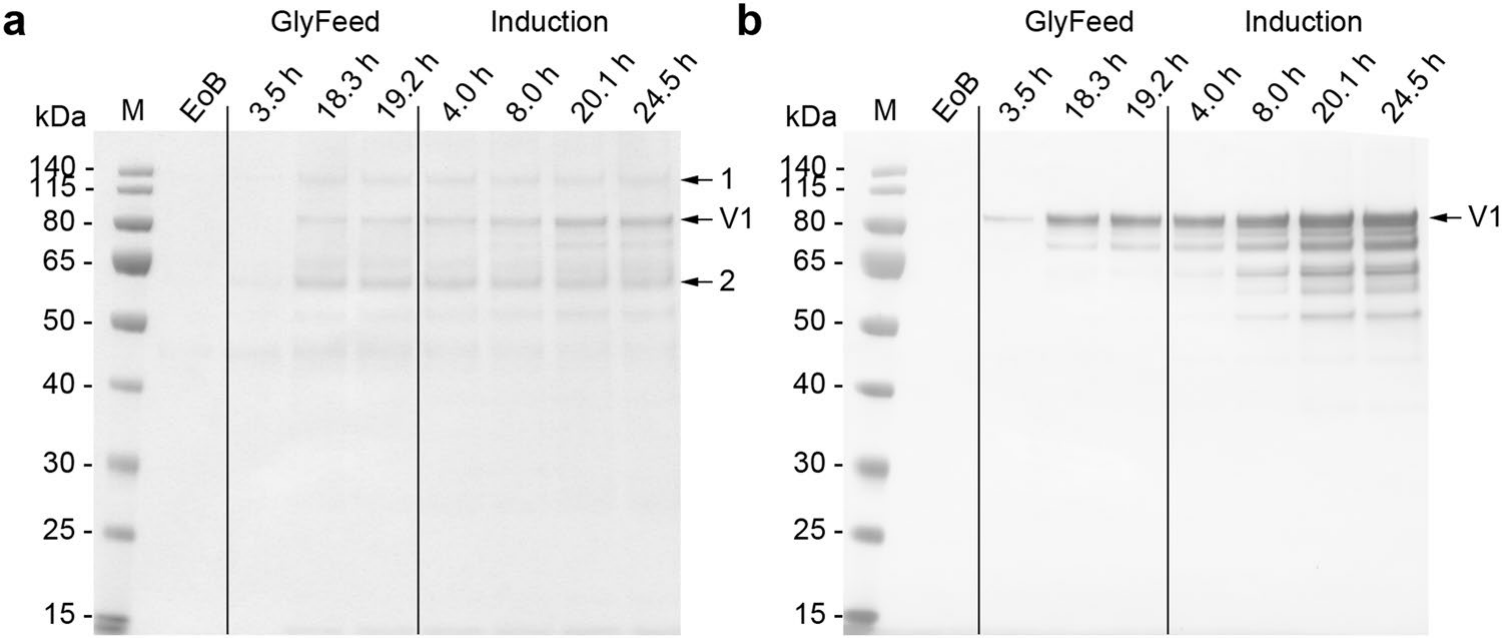
Analysis of fermentation supernatants during the three-phase fermentation process for the production of VAMAX1. (**a**) Proteins in 15 µL supernatant were separated by LDS-PAGE and stained with SimplyBlue SafeStain. Numbered arrows indicate two impurities that were more abundant in the supernatant when the three-phase strategy was used instead of the two-phase strategy. (**b**) The proteins separated above were further analyzed by immunoblotting using the reduction-sensitive mAb 4G2. Times above the lanes indicate the elapsed hours of each process phase. M: protein marker. EoB: end of batch-phase. GlyFeed: glycerol fed-batch phase. Numbered arrows: samples analyzed by mass spectrometry. V1: target protein VAMAX1.

**Figure 4:**
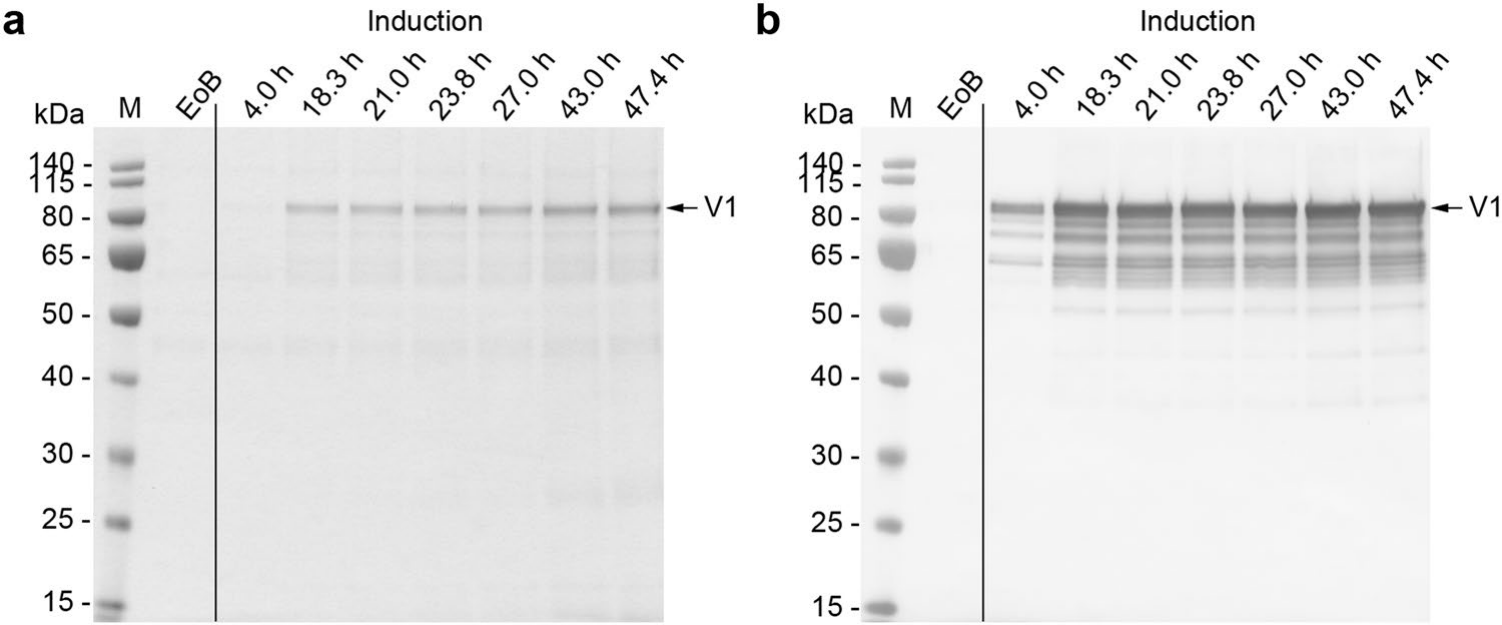
Analysis of fermentation supernatants during the two-phase fermentation process for the production of VAMAX1. (**a**) Proteins in 15 µL supernatant were separated by LDS-PAGE and stained with SimplyBlue SafeStain. (**b**) The proteins separated above were further analyzed by immunoblotting using the reduction-sensitive mAb 4G2. Times above the lanes indicate the elapsed hours of each process phase. M: protein marker. EoB: end of batch-phase. V1: target protein VAMAX1.

**Figure 5:**
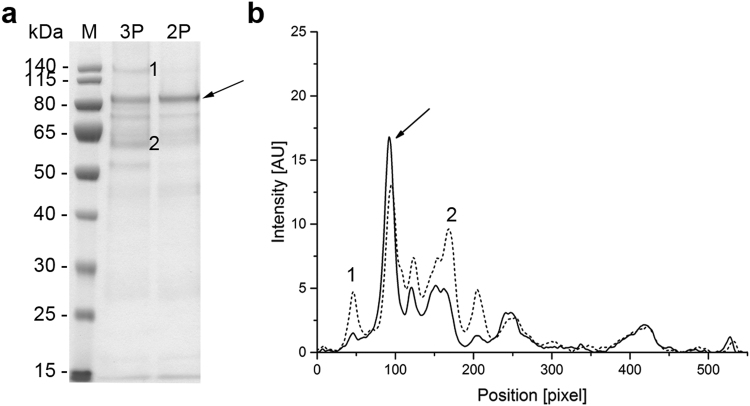
The purity of VAMAX1 in the fermentation supernatant. (**a**) Proteins in 15 µL supernatant taken at the end of the three-phase (3 P) and two-phase (2 P) fermentation strategies were separated by LDS-PAGE and visualized with SimplyBlue SafeStain. (**b**) Densitometric analysis of the separated proteins using AIDA Image Analyzer. Arrows represent the target protein VAMAX1. Numbered bands/peaks represent impurities that were cut from the gel for analysis by mass spectrometry.

**Figure 6:**
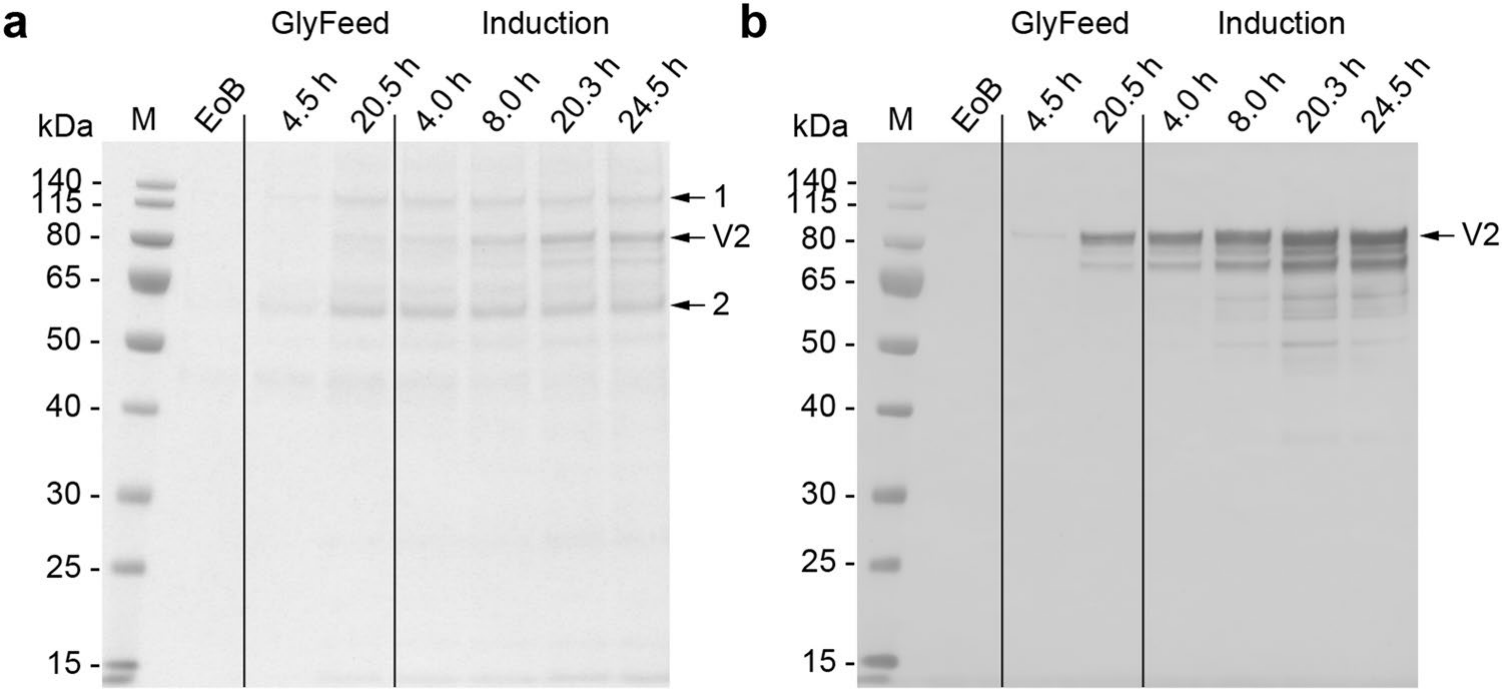
Analysis of fermentation supernatants during the three-phase fermentation process for the production of VAMAX2. (**a**) Proteins in 15 µL supernatant were separated by LDS-PAGE and stained with SimplyBlue SafeStain. Numbered arrows indicate two impurities that were more abundant in the supernatant when the three-phase strategy was used instead of the two-phase strategy. (**b**) The proteins separated above were further analyzed by immunoblotting using the reduction-sensitive mAb 4G2. Times above the lanes indicate the elapsed hours of each process phase. M: protein marker. EoB: end of batch-phase. GlyFeed: glycerol fed-batch phase. Numbered arrows: samples analyzed by mass spectrometry. V2: target protein VAMAX2.

**Figure 7:**
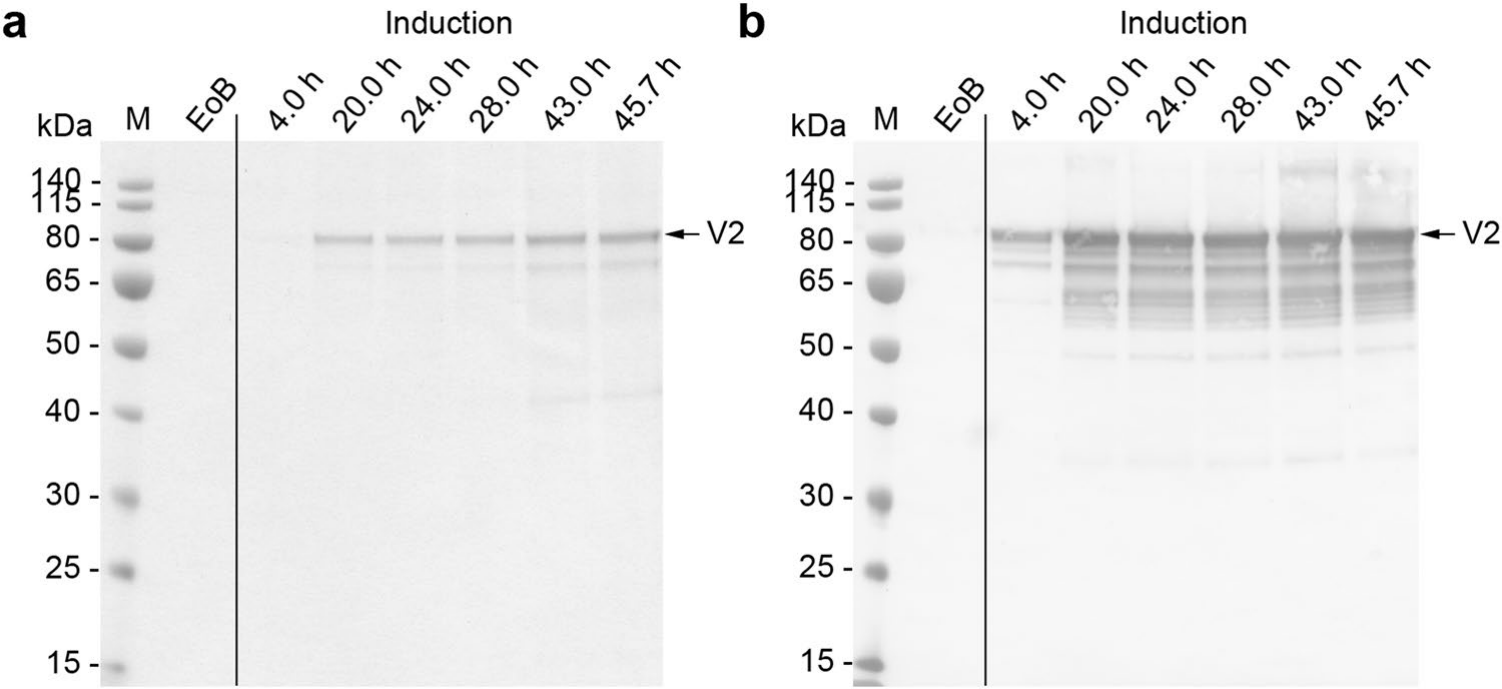
Analysis of fermentation supernatants during the two-phase fermentation process for the production of VAMAX2. (**a**) Proteins in 15 µL supernatant were separated by LDS-PAGE and stained with SimplyBlue SafeStain. (**b**) The proteins separated above were further analyzed by immunoblotting using the reduction-sensitive mAb 4G2. Times above the lanes indicate the elapsed hours of each process phase. M: protein marker. EoB: end of batch-phase. V2: target protein VAMAX2.

Because the methanol concentration was kept constant during the induction phase, the cells were forced into an oxygen-limited expression mode as soon as the maximum OTR was reached, which occurred 12–14 h post-induction. Despite the recommendation to keep the DOT above 20% throughout the fermentation process^13^ because oxygen limitation can negatively affect transgene expression^2^, we did not observe any change in the productivity of the cells or the quality of the recombinant protein in the supernatant when the cells were subjected to oxygen-limited conditions (Figs 3, 4, 5, 6 and 7). This was in agreement with earlier reports where oxygen limitation had no effect^22,38^ or even increased the productivity^39,40^. The higher productivity achieved with the *AOX* promoter and an unlimited methanol supply seems to correlate with the higher AOX activity observed when comparing unlimited and limited methanol induction strategies^39,41^. The latter one also may encourage the loss of cell integrity and/or loss of product due to degradation^40,41^.

### Product yield

Cell growth was reproducible and independent of the vaccine candidate, but it was affected by the fermentation strategy. In contrast, product formation appeared to be affected by both the vaccine candidate and the fermentation strategy. In the three-phase process, the formation of both VAMAX1 and VAMAX2 was already observed during the glycerol fed-batch phase, which can be attributed to the derepression of the *AOX1* promoter^42,43^. In the subsequent induction phase of the three-phase process, VAMAX1 was produced more efficiently than VAMAX2, with the latter reaching only 61% of the final VAMAX1 product concentration (Table 1). The specific productivity of the vaccine candidates therefore differed substantially when the three-phase strategy was used, whereas the productivity was similar for both candidates in the two-phase process (a specific productivity of ~0.019 mg g^−1^ h^−1^) resulting in a productivity increase of 1.7-fold for VAMAX1 and 2.7-fold for VAMAX2 (Table 1).

Although the total process time was similar in both strategies, neither the product concentration nor the specific productivity allowed a direct comparison of process efficiency because the concentration did not take into account differences in final fermentation volume and WCW. Although the latter is encompassed by the specific productivity, the total process time is not considered in the WCW. Therefore, we also compared the space-time yields, first for the induction phase alone and then for the entire process.

Considering the induction phase alone, the VAMAX1 space-time yield was 1.2-fold higher in the three-phase strategy although the concentration of VAMAX1 was 1.6-fold higher in the two-phase process. This reflected the induction time during the two-phase process, which was 1.8-fold longer than that in the three-phase strategy. However, the overall space-time yield was the most relevant factor for the evaluation of process performance, and the two-phase process achieved a 1.5-fold increase over the three-phase strategy resulting in the observed increase in product concentration (Table 1).

The two-phase process already performed better than the three-phase strategy when the induction phase was considered in isolation, achieving a 1.3-fold increase in the space-time yield of VAMAX2. This was improved further when comparing the total process time for both strategies. Here, the two-phase strategy resulted in a 2.5-fold increase in the overall space-time yields for VAMAX2 (Table 1).

The two-phase strategy therefore achieved a considerable increase in the space-time yield for both vaccine candidates even though the cell densities at the time of induction were 76% lower than recommended in the standard protocol^13^. Product yields are generally expected to correlate with the absolute number of cells^44^. This was not the case in our processes, and one explanation for the higher yields despite a lower cell density at the time of induction could be the 4-fold higher growth rate during the production phase of the two-phase process compared to the three-phase process. Correlations have been reported by other groups, albeit not necessarily following a linear function of growth rate and productivity^45–47^. As discussed above, the faster growth rate and thus higher metabolic activity in the pre-induction phase may have facilitated faster adaption to methanol resulting in a higher growth rate during the induction phase of the two-phase process. Thus, we speculate that the pre-induction rate indirectly or directly affects the productivity, as previously suggested for the intracellular expression of *Lateolabrax japonicas* growth hormone in *P*. *pastoris* ^33^. As seen in processes using *E*. *coli* ^48,49^, there was a correlation between the pre-induction growth rate and the production rate during the induction phase.

### Product purity in the supernatant

We investigated the effect of the fermentation strategy on product purity in the supernatant by analyzing the protein profile in the supernatant by LDS-PAGE followed by staining with Coomassie Brilliant Blue. We clearly saw some product-related fragments recognized by the reduction-sensitive monoclonal antibody (mAb) 4G2 in the supernatant from all fermentations (Figs 3, 4, 5, 6 and 7). All VAMAX1 fragments that were detected in the fermentation broth from the three-phase strategy were also found from the broth of the two-phase strategy. This was also the case for VAMAX2, hence the fragment pattern was independent of the strategy. We only observed a slight difference in the intensity distribution of certain fragments when comparing the processes, especially those in the 50–65 kDa size range. Such differences were observed for both VAMAX1 (Figs 3 and 4) and VAMAX2 (Figs 6 and 7).

To assess whether the product-related fragments reflected proteolytic activity in the fermentation broth, we plotted the fragment to full-length ratio throughout the induction phase for a fragment that was distinctly visible following both Coomassie and the western blot analysis (Supplementary Fig. S2a). In addition to a notable fluctuation in the ratio, there was a trend towards a higher fragment/full-product ratio in all fermentations (Supplementary Fig. S2b).

The potential for proteolytic activity was investigated further by testing product stability in the fermentation broth after an initial solid-liquid separation step with a CARR Powerfuge P6. The cell-reduced supernatant (OD_600_ = 2.48 ± 0.01, n = 3) was stored at room temperature and samples were taken directly after centrifugation and at certain time intervals after centrifugation for 10 h (Supplementary Fig. S3a). As above, we observed only a negligible decrease in product concentration over time, i.e. −0.26% loss per hour during the first filtration experiment and −0.24% loss per hour during the second filtration experiment (Supplementary Fig. S3b).

Taken together, the negligible increase in the fragment/full-product ratio and the negligible loss of product during filtration suggest there is minimal proteolytic activity in the fermentation broth, far too low to explain e. g. the >40% fragment/full-product ratio during the three-phase fermentation of VAMAX2 (Supplementary Fig. S2b). Protease-driven degradation can sometimes become a major concern in *P*. *pastoris* fermentations^26,50,51^ and seems to correlate with certain fermentation parameters such as pH, temperature^26,52,53^, and induction time^54^. We assume that proteolytic activity caused the fragmentation of VAMAX1 and VAMAX2, but this must have occurred before the product was secreted to the fermentation broth, e.g. via co-expression/co-secretion degradation as observed for our earlier malaria vaccine candidate PIMP^20^. We were able to substantially reduce the degradation of PIMP by removing the KEX2 consensus motif, which is recognized by a Golgi-located protease^55^.

In addition to product-related fragments, we also detected three weak protein bands with molecular masses of ~46, ~60 and 135 kDa at the end of the batch phase in both the three-phase process (Fig. 3a) and the two-phase process (Fig. 4a) for the production of VAMAX1. These proteins were not detected by mAb 4G2 (Figs 3b and 4b). These data, combined with the tight regulation of the *AOX1* promoter which should prevent the expression of VAMAX1 at that stage of each process^56–58^, indicated that the three bands probably represented contaminating host cell proteins.

In the three-phase strategy, the concentration of these three proteins increased during the glycerol fed-batch phase and then reached a plateau (60 and 135 kDa proteins) or declined slightly (46 kDa protein) throughout the induction phase (Fig. 3a). In contrast, only moderate levels of all three proteins accumulated during the two-phase process (Fig. 4a). Accordingly, densitometric analysis of the Coomassie-stained protein bands from the supernatant taken at the end of the fermentation revealed a strategy-dependent difference in product purity (Fig. 5a). VAMAX1 represented only 22% of the proteins in the supernatant from the three-phase process, but 30% of the proteins in the supernatant from the two-phase process (Fig. 5b). As well as this difference in purity, the quantity of impurities in the supernatant at the end of the process was similar for both strategies: 63 mg L^−1^ for the three-phase process and 65 mg L^−1^ for the two-phase process. The proportion of host cell protein represented by the tree specific bands (46, 60 and 135 kDa) was slightly higher in the three-phase process (34%) compared to two-phase strategy (30%).

Similar results were observed for the production of VAMAX2. We detected three proteins with molecular masses of ~46, 60 and 135 kDa at the end of the batch phase during the three-phase process (Fig. 6a) that were not detected by mAb 4G2 (Fig. 6b). As observed for VAMAX1, these proteins accumulated during the glycerol fed-batch phase, and then reached a plateau (60 and 135 kDa proteins) or declined slightly (46 kDa protein) throughout the induction phase.

**Figure 8:**
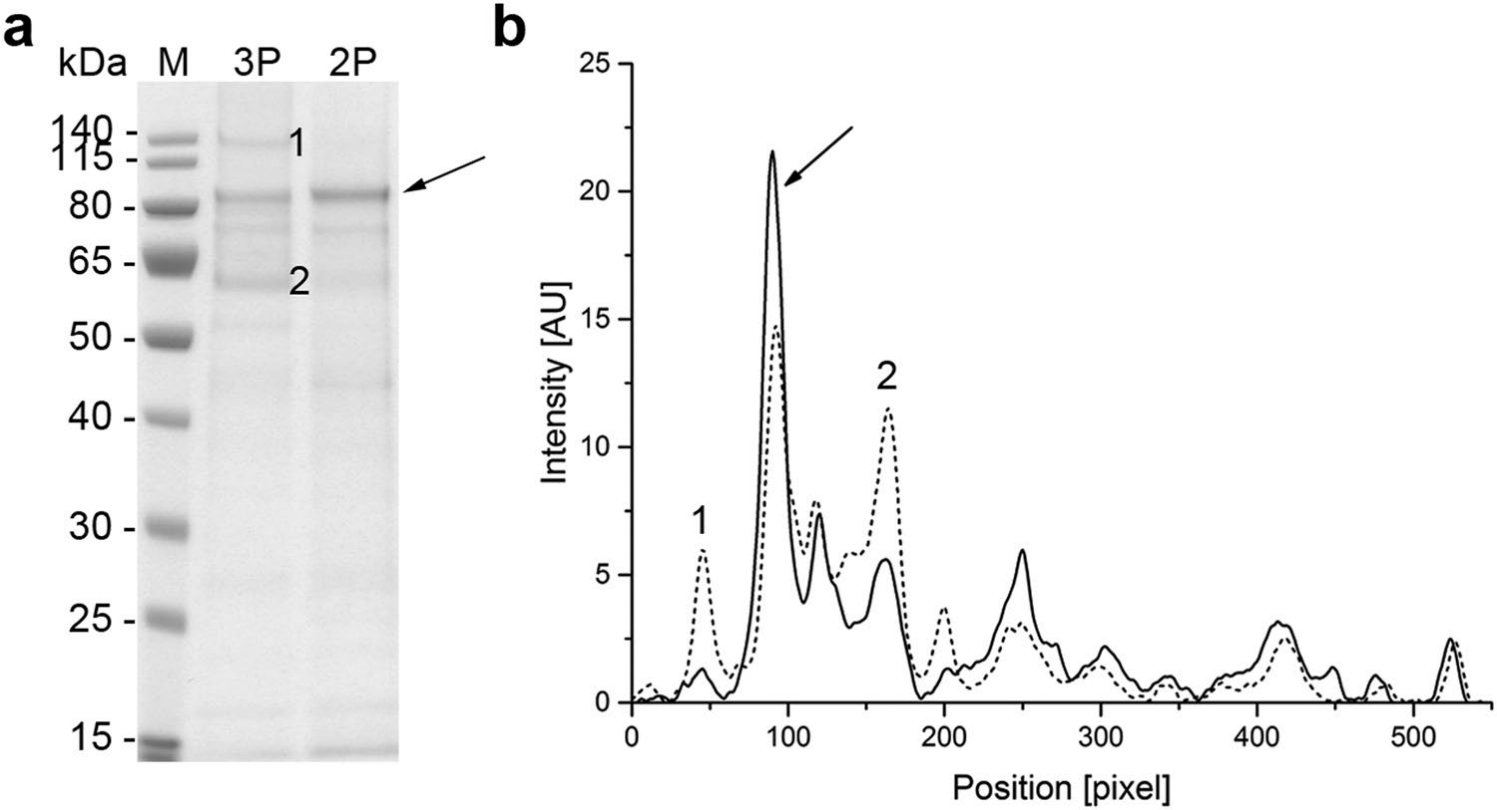
The purity of VAMAX2 in the fermentation supernatant. (**a**) Proteins in 15 µL supernatant taken at the end of the three-phase (3 P) and two-phase (2 P) fermentation strategies were separated by LDS-PAGE and visualized with SimplyBlue SafeStain. (**b**) Densitometric analysis of the separated proteins using AIDA Image Analyzer. Arrows represent the target protein VAMAX2. Numbered bands/peaks represent impurities that were cut from the gel for analysis by mass spectrometry.

In the two-phase process, the three impurities were only detected as faint bands in the later part of the induction phase (Fig. 7a). The two-phase process therefore outperformed the three-phase process in terms of product purity in the supernatant (Fig. 8a). VAMAX2 represented only 19% of the proteins detected in the supernatant from the three-phase process, but 30% of the proteins detected in the supernatant from the two-phase process (Fig. 8b). Although the purity of VAMAX2 was higher in the two-phase strategy, the quantity of impurities in the supernatant at the end of fermentation was considerably lower in the three-phase process (47 mg L^−1^ compared to 62 mg L^−1^). Furthermore, the proportion of host cell protein represented by the three specific bands (46, 60 and 135 kDa) differed according to the fermentation strategy. In the three-phase process, they accounted for 44% of impurities whereas in the two-phase process, they only accounted for 32%.

The 60 and 135 kDa proteins in particular affected the strategy-dependent differences in product purity at the end of fermentation because they accumulated predominantly during the glycerol fed-batch phase, which was omitted in the two-phase process. We therefore analyzed both protein bands by mass spectrometry (Figs 5a and 8a, bands 1 and 2) and tested peptide hits against both the known VAMAX sequences and the NCBInr database for fungi. Peptides representing VAMAX1 and VAMAX2 sequences were found in all samples (Supplementary Table S1). VAMAX peptides were also detected in the 60 kDa protein band (Figs 5a and 8a, band 2) which can be explained by the results of the immunoblot analysis, in which mAb 4G2 recognized product-related fragments with a similar molecular mass. The 135 kDa protein (Figs 5a and 8a, band 1) was also product related based on the mass spectrometry data, but it was not recognized by immunoblot using the reduction-sensitive mAb 4G2 (Figs 3b and 6b) and thus clearly lacked the corresponding binding site. Given the lack of a binding site for mAb 4G2 and the low sequence coverage of 5.9% for VAMAX1 and 12.4% for VAMAX2 in the 135 kDa protein (Supplementary Table S1), this protein may represent a complex of product fragments and/or of such fragments with host cell proteins.

Given the low sequence coverage for VAMAX1 and VAMAX2, we investigated the nature of the 135 kDa protein further and found peptides related to a putative endochitinase (accession number gi|328352741; Supplementary Table S1). Peptides from the same endochitinase, which has an estimated molecular mass of 70 kDa, were also found in the 60 kDa protein in the VAMAX2 fermentation (Fig. 6a, Supplementary Table S1), supporting the hypothesis that the 135 kDa protein is some kind of protein complex.

The NCBInr database yielded hits for two other proteins (Supplementary Table S1) found in both the 135 and 60 kDa protein bands from the VAMAX2 fermentation (Fig. 6a). The first was a putative glucanase (gi|254564921), and the second was a protein of the SUN family (gi|254570078). For all three putative host cell proteins, we found homologous database entries for *S*. *cerevisiae* (Supplementary Data S1) using the UniProt BLAST tool against the UniProtKB database. All of the *S*. *cerevisiae* homologs possess glucosidase hydrolase activity and appear to act as glucanases that are involved in cell separation^59–61^. The involvement of these *S*. *cerevisiae* proteins in cell growth would explain why the 135 and 60 kDa protein bands were more prominent in the three-phase process, where they accumulated mainly during the growth-dedicated glycerol fed-batch phase. The same proteins also accumulated in the two-phase process but at a lower concentration, which may correlate with the 1.3-fold lower cell density in this strategy. Our data therefore suggest that the 135 and 60 kDa proteins represent host cell impurities that increase with the number of cells formed during cultivation.

## Conclusion

For the production of two different malaria vaccine candidates (VAMAX1 and VAMAX2), we switched from the recommended *P*. *pastoris* fermentation protocol that comprises three main stages (batch, glycerol fed-batch, induction) to a simplified process consisting of a batch phase directly followed by the induction phase. Although the cell density at the time of induction in the two-phase strategy was 4-fold lower than that achieved using the standard protocol, the space-time yield increased by 1.5-fold for VAMAX1 and 2.5-fold for VAMAX2. We concluded that productivity during the induction phase was influenced not only by the number of cells but also directly/indirectly by the growth rate during the preceding phase.

The reduced cell density achieved in the two-phase process also had several other beneficial effects: (i) gassing with pure oxygen during the growth phase (batch and glycerol fed-batch phase) was not required and the reactor remained below its maximum OTR; (ii) the reduced cell density can facilitate subsequent downstream processing because less centrifugation time or a smaller filtration area would be required for cell removal; and (iii) the quantity of impurities was reduced, which reduces the pressure on subsequent downstream processing steps. Overall, the benefits of low-cell density fermentation as achieved using the two-phase strategy should have a positive impact on the scalability of the process.

## Materials and Methods

### Vaccine candidate design and cloning

The design and cloning of constructs VAMAXl and VAMAX2 and the selection of recombinant *P*. *pastoris* clones has been described previously^21^. The two constructs were inserted into a *P*. *pastoris* expression vector comprising the methanol-inducible *AOX1* promoter and *AOX1* terminator to control transgene expression. The expression cassette also featured the native *S*. *cerevisiae* α-mating factor secretion sequence, a pUC origin of replication, and a Zeocin resistance gene (*Sh ble*) controlled by the *S*. *cerevisiae TEF1* and synthetic *EM7* promoters and the *S*. *cerevisiae CYC1* terminator. All cloning steps were confirmed by DNA sequencing.

### Transformation and screening of *P*. *pastoris*

The transformation, cultivation and screening of *P*. *pastoris* were carried out as previously described^20^. Supernatants were screened for the secretion of the recombinant proteins by dot blot using the *Pf*s25-specific murine mAb 4B7 obtained from the Malaria Research and Reference Reagent Resource Center (MR4). For each construct, one colony representing a clone with high expression was used to generate a master cell bank (MCB) according to good manufacturing practice (GMP). All clones were of the native methanol utilization phenotype (Mut^+^).

### Pre-culture preparation

The inoculum was prepared from a freshly-thawed vial of the MCB. After thawing to room temperature, 150 µL of the MCB stock was transferred to a 500-mL bottom-baffled shake flask containing 150 mL YSG medium (10 g L^−1^ yeast extract, 20 g L^−1^ soy peptone, 10 g L^−1^ glycerol). The cells were grown for 22 h at 25 °C and 170 rpm.

### Fed-batch fermentation

Fed-batch fermentations were carried out in a refitted BioPilot 40 stirred tank reactor (Applikon, Delft, Netherlands) with a working volume of 30 L at an H/D ratio of 2. The reactor was equipped with three six-blade Rushton turbines. All cultivations were initiated with 15 L mBSM^20^. After *in situ* sterilization, the pH was adjusted to 6.0 with 25% (m/m) ammonia, followed by the aseptic addition of 20 mL of a 10% (v/v) Struktol solution (Struktol J673, Schill + Seilacher “Struktol” GmbH, Hamburg, Germany) and the addition of a mPTM solution^20^. The volume of the mPTM solution was calculated based on the expected total amount of carbon source for all fermentation steps:

1 $${V}_{mPTM,total}={V}_{mPTM,batch}+{V}_{mPTM,gf}+{V}_{mPTM,ind}$$

given that

2 $${V}_{mPTM,batch}=0.4\frac{m{L}_{mPTM}}{{g}_{glycerol,batch}}\times {m}_{glycerol,batch}$$

3 $${V}_{mPTM,gf}=0.048\frac{m{L}_{mPTM}}{{g}_{glycerol,feed}}\times {m}_{glycerol,feed}$$

4 $${V}_{mPTM,ind}=30\frac{m{L}_{mPTM}}{k{g}_{methanol}}\times {m}_{methanol}$$

where m_glycerol,batch_ is the absolute mass of glycerol (100% [m/m]) in mBSM, m_glycerol,feed_ is the expected total mass of glycerol to be provided during the glycerol fed-batch phase (*gf*), and m_methanol_ is the expected total mass of methanol to be provided during the induction phase (*ind*).

After inoculation of the bioreactor with 150 mL of pre-culture, the fermentation was started with the following initial parameters: growth temperature = 28 °C, aeration rate = 15 L min^−1^, headspace gauge pressure = 1 bar, agitation rate = 350–600 rpm, DOT = 30%, and pH = 6.0 (controlled by the addition of 25% [m/m] ammonia). When a sharp rise in DOT indicated the depletion of the batch glycerol after 22–24 h, the fermentation was continued according to either the three-phase or two-phase strategy described below.

### Three-phase strategy

In the three-phase strategy, the batch phase was followed by the addition of a 50% (m/m) glycerol solution using a DOT-based closed-loop control with a set point of 30% and a fixed agitation rate of 530 rpm. The process was switched to the induction phase (third phase) after the addition of 2500 g glycerol solution. The temperature was shifted to 25 °C and a bolus of 2.5 mL L^−1^ methanol was added to the cell broth. The broth volume was calculated by taking all added solutions and removed sample volumes into account. The methanol concentration was kept constant at 2.5 mL L^−1^ for 24 h (see methanol feed, below) such that the total amount of added methanol was 1.0–1.1 kg. During this phase, the DOT was controlled at a set point of 30% by increasing the agitation rate but it was allowed to drop to 0% at the maximum speed of 600 rpm ultimately switching to an oxygen-limited induction mode.

### Two-phase strategy

The two-phase strategy comprised the batch phase described above and an induction phase. The induction phase (second phase) was initiated and controlled as described for the three-phase strategy. Following the addition of 2700 g methanol, the process was stopped as soon as the methanol concentration dropped to 0.0 mL L^−1^.

### Methanol feed

An ALCOSENS probe combined with an ACETOMAT N II control system (Heinrich Frings GmbH, Rheinbach, Germany) was used to monitor the methanol concentration during the induction phase. The probe was calibrated before each fermentation run at three different methanol concentrations (0.5, 2.5 and 5.0 mL L^−1^) using deionized water at 25 °C (induction temperature). Based on these data, the probe was recalibrated at the beginning of the induction phase by a parallel shift of the calibration curve using the resistance readout of the 2.5 mL L^−1^ methanol bolus (see three-phase strategy, above).

The signal of the recalibrated probe was processed in a programmed proportional and integral (PI) controller using the control and data acquisition software BioXpert XP v3.72 (Applikon Biotechnology) to control the methanol feed rate via a Masterflex P/S peristaltic pump (model 1300–3600, Thermo Fisher Scientific, Waltham, MA, USA).

### Cell concentration

The cell concentration was monitored by measuring the optical density at 600 nm. To estimate the WCW, 1.5 mL culture broth was centrifuged in a pre-weighed test tube at 16,100 × *g* for 2 min. The supernatant was collected for further analysis and the mass of the remaining cell pellet was determined. The DCW was estimated by drying the cell pellet at 60 °C until a constant weight was achieved.

### LDS-PAGE and western blot analysis

Samples were mixed with 4-fold concentrated LDS sample buffer (NuPage, Thermo Fisher Scientific) and loaded onto commercial 10% (m/v) isocratic Bis-Tris polyacrylamide gels (NuPage). The proteins were separated at 200 V for 50 min under non-reducing conditions using MOPS SDS running buffer (NuPage). After electrophoresis, the proteins were either stained with Coomassie (SimplyBlue SafeStain, Thermo Fisher Scientific) or blotted onto a nitrocellulose membrane (0.2 µm) at 30 V for 1 h, followed by blocking with 5% (m/v) skimmed milk dissolved in phosphate buffered saline (PBS) supplemented with 0.05% (v/v) Tween-20 (PBS-T). The blotted proteins were probed with the reduction-sensitive mAb 4G2^62^ at a concentration of 0.86 µg mL^−1^. Bands were visualized using an alkaline phosphatase-conjugated polyclonal goat-anti-rat antibody (H + L) (Jackson ImmunoResearch, West Grove, PA, USA) combined with nitroblue tetrazolium and 5-bromo-4-chloro-3-indolyl-phosphate solution (NBT/BCIP; Roth, Karlsruhe, Germany). The membranes were washed three times for 3 min with PBS-T between incubation steps. Images of stained gels and western blots were acquired with a CanoScan 5600 F (Canon Inc., Tokyo, Japan) and Photoshop Elements v5.0.2 (Adobe Systems, San Jose, CA, USA) at a resolution of 600 dpi.

### Mass spectrometry

In-gel protein digestion was carried out as previously described^63^. The resulting peptides were analyzed using a nanoHPLC (UltiMate 3000 HPLC system, LC PAcking, Dionex, Idstein, Germany) coupled to an amaZon electron-transfer dissociation (ETD) ion trap mass spectrometer (Bruker Daltonics, Bremen, Germany) with an electron spray ionization (ESI) nanosprayer. MS/MS spectra were screened for peptides of interest using the search engine Mascot Search v2.3.01 (Matrix Science Ltd, London, UK). The detailed MS data are provided in Supplementary Text S1.

### Product quantification

The quantity of the target protein in the culture supernatant was determined by densitometric in-gel quantification. Product standards (purity > 97%) were included on each gel, ranging from 50 to 300 ng per lane. The scanned gels were evaluated using AIDA Image Analyzer software (Raytest, Elysia Germany GmbH, Straubenhardt, Germany) and a polynomial quantification curve (second order) was generated from the background-corrected integral of the product standards. To determine the product concentration in the supernatant, the background-corrected integral of the target band in each sample lane was plotted on the standard curve and divided by the absolute sample volume applied to the lane.

### Statistics

Fermentations were run as single biological experiments. Analytical data such as cell density and product concentration are represented as technical triplicates.

## Electronic supplementary material

Supplementary data
