# Supplementary material for: Improvement of a fermentation process for the production of two PfAMA1-DiCo-based malaria vaccine candidates in Pichia pastoris
Source: Sci Rep. 2017 Sep 20;7:11991. doi: 10.1038/s41598-017-11819-4 (PMC5607246; doi:10.1038/s41598-017-11819-4)
Supplement: Supplementary file 1 — Supplementary data [file 41598_2017_11819_MOESM1_ESM.pdf]

Title: Improvement of a fermentation process for the production of two *Pf*AMA1-DiCo-based malaria vaccine candidates in *Pichia pastoris*

Authors: Robin Kastilan[1], Alexander Boes[1], Holger Spiegel[1,*], Nadja Voepel[1], Ivana Chudobová[1], Stephan Hellwig[1], Johannes Felix Buyel[1,2], Andreas Reimann[1], Rainer Fischer[2,3]

Supplementary Text S1 - Mass spectrometry

Proteins were reduced, alkylated and digested in the polyacrylamide gel using trypsin (Promega, Mannheim, Germany) as previously described[1]. The resulting peptides were analyzed by nanoHPLC using an UltiMate 3000 HPLC system (LC PAcking, Dionex, Idstein, Germany) coupled to an amaZon ETD ion trap mass spectrometer (Bruker Daltonics, Bremen, Germany) with an ESI nanosprayer. The nanoHPLC system and the ion trap mass spectrometer were controlled using Bruker Compass HyStar v3.2 SR2 software. The liquid chromatography system comprised a reversed-phase pre-column (LC PAckings, Dionex) for sample desalting and a 15-cm PepMap 100 reversed-phase C18 column, 75 µm inner diameter (LC PAckings, Dionex), for peptide fractionation. The peptides were separated using a 45-min linear gradient from 96% (v/v) solution A (2% (v/v) acetonitrile, 0.1% (v/v) formic acid in high-purity water) and 4% (v/v) solution B (98% (v/v) acetonitrile, 0.1% (v/v) formic acid in high-purity water) to 50% (v/v) solution A and 50% (v/v) solution B at a flow rate of 300 nL min^{-1}. The electrospray was operated in positive ion mode with a spray voltage of –4000 V and 10 psi gas pressure. The end plate offset of the mass spectrometer was set to –500 V and Proteomics AutoMSMS Alternating Spectra CID-ETD Bruker trapControl v7.0 was used for data acquisition. Raw data files were evaluated using Compass DataAnalysis v4.0 SR5 software with embedded search engine Mascot Search v2.3.01 (Matrix Science Ltd, London, UK). The spectra were searched against the NCBInr fungi database and the cRAP database (including VAMAX1 and VAMAX2 sequences) using the following parameters: enzyme = trypsin, up to one missed cleavage permitted, no fixed modifications, variable modifications allowed = carbamidomethyl (C), oxidation (M) and propionamide (C), mass tolerance for precursor ion ±0.3 Da and fragment ion ±0.3 Da.

Supplementary Figure S1

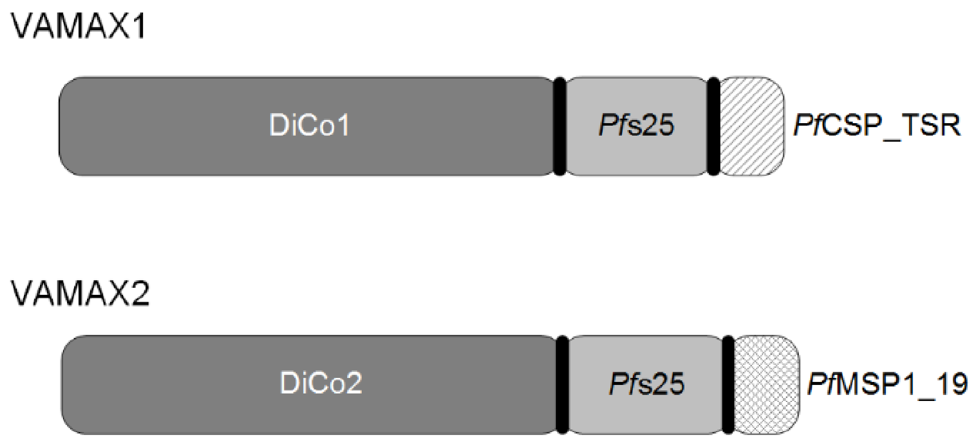

Figure S1 Schematic representation of the VAMAX1 and VAMAX2 fusion antigens. DiCo1 and DiCo2 = diversity-covering variants of *Pf*AMA1 (dark gray); *Pf*s25 = *Plasmodium falciparum* D7 sexual-stage surface antigen *Pf*s25 (light gray); *Pf*CSP_TSR = thrombospondin-related region from *P. falciparum* 3D7 circumsporozoite protein *Pf*CSP (hatched); *Pf*MSP1_19 = C-terminal 19 kDa fragment of the merozoite surface protein 1 from *P. falciparum* strain FUP (cross-hatched).

Supplementary Figure S2

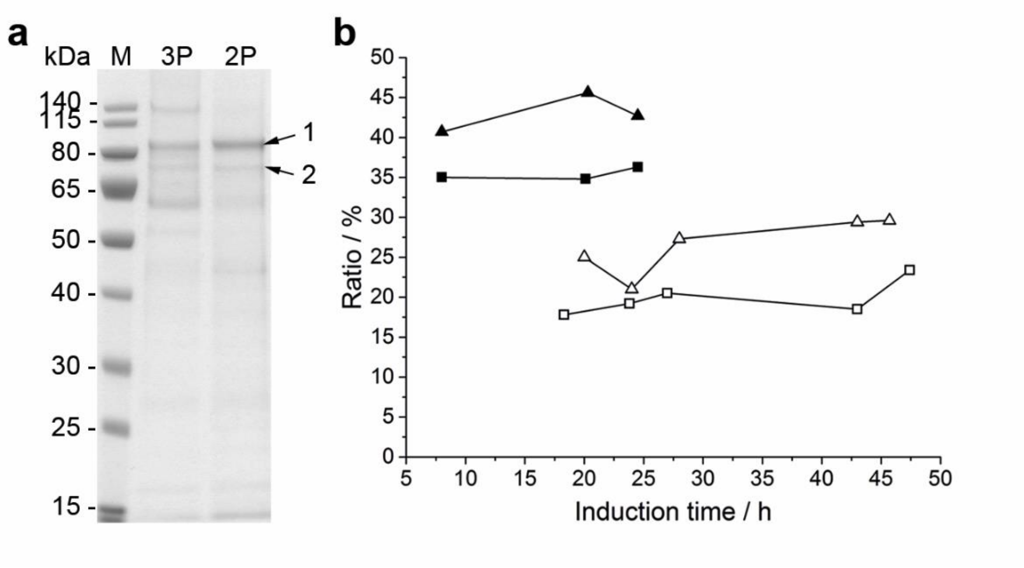

Figure S2 Investigation of potential proteolytic activity in the fermentation broth during the course of induction. **(a)** Samples of the supernatant during the induction phase of both strategies (three-phase = 3P, two-phase = 2P) were analyzed by LDS-PAGE and Coomassie-staining followed by the assessment of the background-corrected intensity of protein band 1 (VAMAX1) and protein band 2 (degradation product of VAMAX1) using AIDA Image Analyzer software (Raytest). Representative samples shown here from the end of the fermentation runs. M: marker. **(b)** To detect potential proteolytic activity in the fermentation broth the intensity ratio of band 1 to band 2 was plotted during the induction phase. Filled symbols: three-phase strategy. Open symbols: two-phase strategy. Squares: VAMAX1 fermentations. Triangles: VAMAX2 fermentations.

Supplementary Figure S3

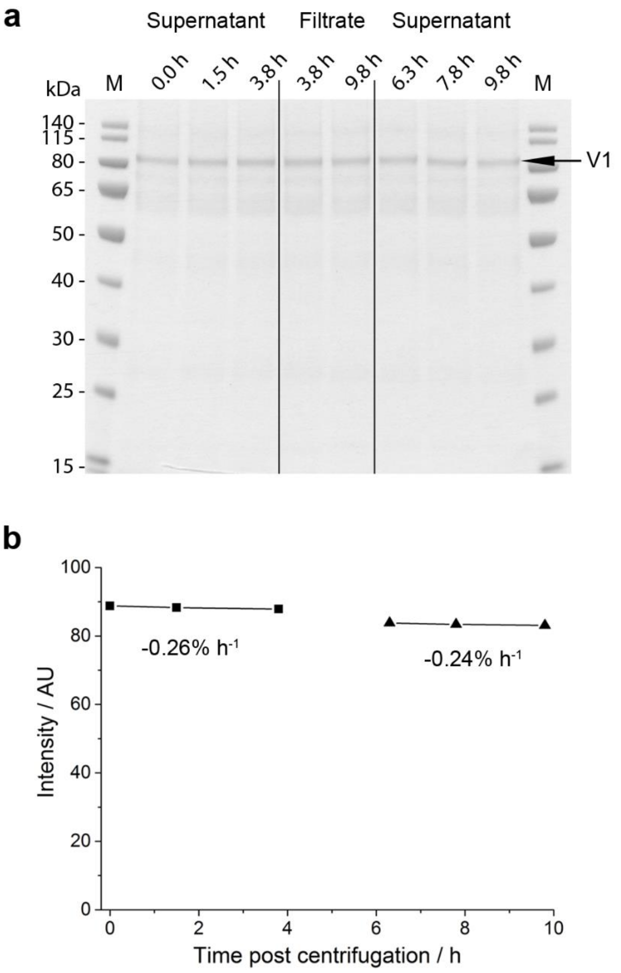

Figure S3 Stability of VAMAX1 (V1) in the supernatant after solid–liquid separation by centrifugation. At the end of the two-phase process, the cells were semi-continuously separated from the fermentation broth by centrifugation with a CARR Powerfuge P6 at 5,500 × *g* and a feed rate of 0.75 kg min^{-1}. The cell density was considerably reduced to an OD_{600} of 2.48 ± 0.01 (n = 3) in the supernatant. **(a)** The stability testing of VAMAX1 was performed during two consecutive filtration experiments starting directly (0.0 h) and 6.3 h after centrifugation, respectively, at room temperature. Samples of the supernatant were taken at the indicated times post-centrifugation and were analyzed by LDS-PAGE and Coomassie-staining. **(b)** The Coomassie-stained bands of VAMAX1 were evaluated by densitometric analysis using AIDA Image Analyzer software. The background-corrected integral of each band was plotted over time post-centrifugation. During the first filtration experiment, the apparent decrease in band intensity (representing product loss) was –0.26% per hour, calculated using a linear regression function (R^2 = 0.97). For the second filtration experiment, the decrease in band intensity was –0.24% per hour (R^2 = 0.97).

Supplementary Table S1 – Mass spectrometry data

**Candidate / Protein band**	**Accession**	**Protein**	**Score**	**Peptides**	**Sequence coverage**
**VAMAX1**					
**Band 1**	VAMAX1_Dico1_ pfs25FKO_CSP_TSR	VAMAX1	103.1	4	5.9%
	gi|328352741	Endochitinase [*Komagataella phaffii* CBS 7435]	137.0	2	8.0%
**Band 2**	VAMAX1_Dico1_ pfs25FKO_CSP_TSR	VAMAX1	396.1	8	12.9%
**VAMAX2**					
**Band 1**	VAMAX2_Dico2_ pfs25FKO_MSP1_19	VAMAX2	313.2	8	12.4%
	gi|328352741	Endochitinase [*K. phaffii* CBS 7435]	273.0	3	10.0%
	gi|254564921	Cell wall protein with similarity to glucanases [*K. phaffii* GS115]	237.0	4	15.0%
	gi|254570078	Protein of the SUN family (Sim1p, Uth1p, Nca3p, Sun4p) that may participate in DNA replication [*K. phaffii* GS115]	193.0	2	9.0%
**Band 2**	VAMAX2_Dico2_ pfs25FKO_MSP1_19	VAMAX2	539.1	12	21.5%
	gi|254564921	Cell wall protein with similarity to glucanases [*K. phaffii* GS115]	196.0	3	11.0%
	gi|254570078	Protein of the SUN family (Sim1p, Uth1p, Nca3p, Sun4p) that may participate in DNA replication [*K. phaffii* GS115]	171.0	2	9.0%

Supplementary Data S1

Sequences of the putative host cell proteins identified by mass spectrometry (Supplementary Table S1) were used as BLAST queries against the UniProtKB database with the following parameters:

- Threshold e-value: 0.001
- Matrix: auto
- Filtering: none
- Gapped: yes
- Hits: 100

Alignment with an endochitinase from *Saccharomyces cerevisiae* (UniProtKB entry P29029) found by using gi|328352741 (endochitinase [*Komagataella phaffii* CBS 7435]) as a BLAST query:

P29029 CHIT_YEAST - **Endochitinase** Saccharomyces cerevisiae (strain ATCC 204508 / S288c) (Baker's yeast)

E-value: 6.8e-133
Score: 1067
Ident.: 48.0%
Positives : 66.1%
Query Length: 686
Match Length: 562

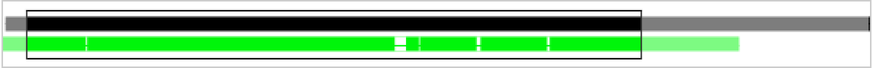

```
F2QUR1 F2QUR1_KOMPC     18      AFDNSAKNNVALYWGQNSAGSQERLSYYCQSDSVDIVLLSFLYIFPANPLGLDFSNACGD      77
                                AFD SA  N+A+YWGQNSAG+QE L+ YC+S   DI LLSFL  FP   LGL+F+NAC D
P29029 CHIT_YEAST       20      AFDRSANTNIAVYWGQNSAGTQESLATYCESSDADIFLLSFLNQFPT--LGLNFANACSD      77

F2QUR1 F2QUR1_KOMPC     78      QFPSGLLKCDTIAEDIQTCQSLGKKVLLSLGGATGTYGFSSDSEAEDFAEVLWDTF-LGG     136
                                 F  GLL C  IAEDI+TCQSLGKKVLLSLGGA+G+Y FS DS+AE FA+ LWDTF  G
P29029 CHIT_YEAST       78      TFSDGLLHCTQIAEDIETCQSLGKKVLLSLGGASGSYLFSDDSQAETFAQTLWDTFGEGT     137

F2QUR1 F2QUR1_KOMPC     137     STDERPFGDSILDGIDYDAENNNPTGYTALSAKLREFYASDPSRTYYIAAAPQCPYPDAS     196
                                   ERPF  +++DG D+D ENNN  GY+AL+ KLR  +A + ++ YY++AAPQCPYPDAS
P29029 CHIT_YEAST       138     GASERPFDSAVVDGFDFDIENNNEVGYSALATKLRTLFA-EGTKQYYLSAAPQCPYPDAS     196

F2QUR1 F2QUR1_KOMPC     197     VGDVLANADVDFVFIQFYNNYCALASTSFNWATWLDYAQNTSPNPNVKLYVGLPGGPTGA     256
                                VGD+L NAD+DF FIQFYNNYC++ S  FNW TWL YAQ  SPN N+KL++GLPG  + A
P29029 CHIT_YEAST       197     VGDLLENADIDFAFIQFYNNYCSV-SGQFNWDTWLTYAQTVSPNKNIKLFLGLPGSASAA     255

F2QUR1 F2QUR1_KOMPC     257     SSGYVG-TDVVKQRIDEIGASSSLGGIMLWDASQGFSNQVDGGNYVDAMKSILNGLGSVD     315
                                 SGY+  T +++  I +I +SSS GGI LWDASQ FSN+++G  YV+ +K++L
P29029 CHIT_YEAST       256     GSGYISDTSLLESTIADIASSSSFGGIALWDASQAFSNELNGEPYVEILKNLL-------     308

F2QUR1 F2QUR1_KOMPC     316     ASTTSSSQAAATSQTTSTLATSISSTPGSSSTVSSSSSLSSSSSPSSSSSSTTIWWTPDT     375
                                   TS+SQ A T  T +T  TS +ST  +S++ +S+S   ++ S +S+ S + +  +P
P29029 CHIT_YEAST       309     ---TSASQTATT--TVATSKTSAASTSSASTSSASTSQKKTTQSTTSTQSKSKVTLSP--     361

F2QUR1 F2QUR1_KOMPC     376     PQSSSSSVAAVSSETSSVLTSSVSTTQNSHGEVDSEGTTLTGTSTIWWTPSEAQSYETSS     435
                                  ++SS++    ++T+  LTSS + T++S G   +E T  +   T   T   +Q    +S
P29029 CHIT_YEAST       362     --TASSAIKTSITQTTKTLTSS-TKTKSSLGTTTTESTLNSVAITSMKTTLSSQ---ITS     415

F2QUR1 F2QUR1_KOMPC     436     LSSVSSIPTGNKDVSSILVITDVTDSLT-STKESSDSALTISTSLSSSPSLADSSRDGET     494
                                 + V+    T    VSS  + T +T +L+ +TK SS  +L  +T+ + SP+   +S  G T
P29029 CHIT_YEAST       416     AALVTPQTTTTSIVSSAPIQTAITSTLSPATKSSSVVSLQTATTSTLSPTTTSTS-SGST     474

F2QUR1 F2QUR1_KOMPC     495     STVVQVTSST                                                        504
                                S+    + ST
P29029 CHIT_YEAST       475     SSGSTSSDST                                                        484
```

Alignment with a putative family 17 glucosidase SCW10 from *S. cerevisiae* (UniProtKB entry Q04951) found by using gi|254564921 (cell wall protein with similarity to glucanases [*K. phaffii* GS115]) as a BLAST query:

Q04951 SCW10_YEAST - **Probable family 17 glucosidase SCW10** Saccharomyces cerevisiae (strain ATCC 204508 / S288c) (Baker's yeast)

E-value: 3.5e-124
Score: 961
Ident.: 55.5%
Positives : 67.8%
Query Length: 348
Match Length: 389

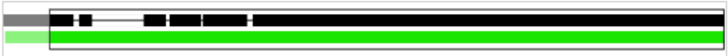

```
C4QVL7 C4QVL7_PICPG   26    HQHDKRGVVVVTK----TIVVDG-----------------------------STVEATAA    52
                            H+H+KR VV  T     T+VV G                             ST+E T +
Q04951 SCW10_YEAST    24    HKHEKRDVVTATVHAQVTVVVSGNSGETIVPVNENAVVATTSSTAVASQATTSTLEPTTS    83

C4QVL7 C4QVL7_PICPG   53    AQV---QEHAETFAESTPSAVV--SSSSAPSSASSASAPASSGSFSA----GTKGVTYSP   103
                            A V   Q+   T   S  ++ V  S+SS+PSS+SS S+ ASS + S+    G KG+TYSP
Q04951 SCW10_YEAST    84    ANVVTSQQQTSTLQSSEAASTVGSSTSSSPSSSSSTSSSASSSASSSISASGAKGITYSP   143

C4QVL7 C4QVL7_PICPG   104   YQAGGGCKTAEEVASDLSQLTGYEIIRLYGVDCNQVENVFKAKAPGQKLFLGIFFVDAIE   163
                            Y   G CK+  +VASDL QLTG++ IRLYGVDC+QVENV +AK   QKLFLGI++VD I+
Q04951 SCW10_YEAST    144   YNDDGSCKSTAQVASDLEQLTGFDNIRLYGVDCSQVENVLQAKTSSQKLFLGIYYVDKIQ   203

C4QVL7 C4QVL7_PICPG   164   SGVSAIASAVKSYGSWDDVHTVSVGNELVNNGEATVSQIGQYVSTAKSALRSAGFTGPVL   223
                              V  I SAV+SYGSWDD+ TVSVGNELVN G AT +Q+G+YVSTAKSAL SAG+TG V+
Q04951 SCW10_YEAST    204   DAVDTIKSAVESYGSWDDITTVSVGNELVNGGSATTTQVGEYVSTAKSALTSAGYTGSVV   263

C4QVL7 C4QVL7_PICPG   224   SVDTFIAVINNPGLCDFADEYVAVNAHAFFDGGIAASGAGDWAAEQIQRVSSACGG-KDV   282
                            SVDTFIAVINNP LC+++D Y+AVNAHA+FD   AA  AG W  EQI+RV +ACGG KDV
Q04951 SCW10_YEAST    264   SVDTFIAVINNPDLCNYSD-YMAVNAHAYFDENTAAQDAGPWVLEQIERVYTACGGKKDV   322

C4QVL7 C4QVL7_PICPG   283   LIVESGWPSKGDTNGAAVPSKSNQQAAVQSLGQKIGSSCIAFNAFNDYWKADGPFNAEKY   342
                            +I E+GWPSKGDT G AVPSK+NQ+AA+ S+    GSS   F AFND WK DG +  EKY
Q04951 SCW10_YEAST    323   VITETGWPSKGDTYGEAVPSKANQEAAISSIKSSCGSSAYLFTAFNDLWKDDGQYGVEKY   382

C4QVL7 C4QVL7_PICPG   343   WGILDS                                                         348
                            WGIL S
Q04951 SCW10_YEAST    383   WGILSS                                                         388
```

Alignment with a probable secreted β-glucosidase SUN4 from *S. cerevisiae* (UniProtKB entry P53616) found by using gi|254570078 (Protein of the SUN family [*K. phaffii* GS115]) as a BLAST query:

P53616 SUN4_YEAST - **Probable secreted beta-glucosidase SUN4** Saccharomyces cerevisiae (strain ATCC 204508 / S288c) (Baker's yeast)

E-value: 3.3e-163
Score: 1231
Ident.: 56.4%
Positives : 69.9%
Query Length: 431
Match Length: 420

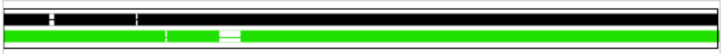

```
C4R2Z5 C4R2Z5_PICPG   1     MKISALTACAVTLAGLA-IAAPAPKPED----CTTTVQKRHQHKRAVAYDYVYVTVTING    55
                            MK+SA T  A +L G + I +  P   D    CTTT    HQHKRAVA  YVY TVT++
P53616 SUN4_YEAST     1     MKLSATTLTAASLIGYSTIVSALPYAADIDTGCTTTAHGSHQHKRAVAVTYVYETVTVDK    60

C4R2Z5 C4R2Z5_PICPG   56    QGETISPTVVAELETVSTPAT--SSSAVATSSAPATTSSIPETTSAQVTTSSVAESTSAQ    113
                             G+T++PT      TV++  T  S S+V  SS+   +SS  E+T    TTSS A++T
P53616 SUN4_YEAST     61    NGQTVTPTSTEASSTVASTTTLISESSVTKSSSKVASSS--ESTEQIATTSSSAQTTLTS    118

C4R2Z5 C4R2Z5_PICPG   114   FTSSTEEASSSVEQTTQSSSSSSPSSSSSSSSSSGSSSGSSSGGIDGDLSWYSGPGNKFQ    173
                              +ST E+S  +              S+S S+S+ S++ S++G I GDL+ +SGP  KF+
P53616 SUN4_YEAST     119   SETSTSESSVPI--------------STSGSASTSSAASSATGSIYGDLADFSGPYEKFE    164

C4R2Z5 C4R2Z5_PICPG   174   DGTIKCSEFPSGNGVVALDHLGFGGWSGLYHPSDTSTGGICEPDTYCSYACQSGMSKTQW    233
                            DGTI C +FPSG GV+ +  L  GGWSG+ + +DTSTGG C+  +YCSYACQ GMSKTQW
P53616 SUN4_YEAST     165   DGTIPCGQFPSGQGVIPISWLDEGGWSGVEN-TDTSTGGSCKEGSYCSYACQPGMSKTQW    223

C4R2Z5 C4R2Z5_PICPG   234   PSEQPDNGVSVGGLYCDSDGYLYRSKKDTDYLCEWGIDVAYVVSEIGKTAAICRTDYPGT    293
                            PS+QP +G S+GGL C  DGYLYRS  DTDYLCEWG+D AYVVSE+    AICRTDYPGT
P53616 SUN4_YEAST     224   PSDQPSDGRSIGGLLC-KDGYLYRSNTDTDYLCEWGVDAAYVVSELSNDVAICRTDYPGT    282

C4R2Z5 C4R2Z5_PICPG   294   ENMVIPTVVSPGGELPLTTVDQSSYYTWRGMKTSAQYYVNDAGVDWTTGCTWGDYGTDYG    353
                            ENMVIPT V  G  LPLT VDQ +YYTW+G+KTSAQYYVN+AG+     C WG   +  G
P53616 SUN4_YEAST     283   ENMVIPTYVQAGDSLPLTVVDQDTYYTWQGLKTSAQYYVNNAGISVEDACVWGSSSSGVG    342

C4R2Z5 C4R2Z5_PICPG   354   NWAPLNFGAGYDNGISYLSLIPNPNNKDSLNYNVKIVAYDDSSVVIGECKYENGSYNGNG    413
                            NWAPLNFGAG  +G++YLSLIPNPNN ++LN+NVKIVA DDSS V GEC YENGS++G G
P53616 SUN4_YEAST     343   NWAPLNFGAGSSDGVAYLSLIPNPNNGNALNFNVKIVAADDSSTVNGECIYENGSFSG-G    401

C4R2Z5 C4R2Z5_PICPG   414   SDGCTVAVNSGKAKFVLY                                              431
                            SDGCTV+V +GKAKFVLY
P53616 SUN4_YEAST     402   SDGCTVSVTAGKAKFVLY                                              419
```

Supplementary Reference:

1. Shevchenko, A., Tomas, H., Havlis, J., Olsen, J.V. & Mann, M. In-gel digestion for mass spectrometric characterization of proteins and proteomes. *Nat. Protoc.* **1**, 2856-60, doi:10.1038/nprot.2006.468 (2006).
